# miR-155-5p in Extracellular Vesicles Derived from Choroid Plexus Epithelial Cells Promotes Autophagy and Inflammation to Aggravate Ischemic Brain Injury in Mice

**DOI:** 10.1155/2022/8603427

**Published:** 2022-02-16

**Authors:** Zhang Yang, Xiaofang Shi, Zidan Gao, Lan Chu

**Affiliations:** ^1^Department of Neurology, Affiliated Hospital of Guizhou Medical University, Guizhou Medical University, Guiyang, China; ^2^Department of Translational Medicine Research Center, Guizhou Medical University, Guiyang, China

## Abstract

Ischemic stroke is a common disease of the central nervous system, and ischemic brain injury (IBI) is its main manifestation. Recently, extracellular vesicles (EVs) have been strongly related to the diagnosis and treatment of IBI. However, the underlying mechanism of their effects remains enigmatic. In the present study, we aimed to study how miR-155-5p plays a role in choroid plexus epithelial (CPE) cell-derived EVs in IBI pathology. We found that miR-155-5p expression was enriched in CPE cell-derived EVs, which were subsequently internalized by neurons, enabling the delivery of miR-155-5p into neurons. An inducible oxygen and glucose deprivation and reoxygenation (OGD/R) cell model was developed to mimic ischemic neuronal injury *in vitro*. miR-155-5p overexpression led to reduced neuron viability, promoted apoptosis, elevated autophagic proteins' expression, and activated NLR family pyrin domain-containing 3- (NLRP3-) related inflammasomes, thereby aggravating OGD-induced neuronal injury. A dual-luciferase reporter assay exhibited that miR-155-5p could inhibit the Ras homolog enriched in brain (Rheb) expression, a mechanism critical for miR-155-5p-mediated neuronal injury. Furthermore, a mouse IBI model was developed using the transient middle cerebral artery occlusion (tMCAO) method. Animal experiments verified that miR-155p delivery via CPE cell-derived EVs aggravated IBI by suppressing Rheb expression. In conclusion, miR-155-5p in CPE-derived EVs can aggravate IBI pathology by suppressing Rheb expression and promoting NLRP3-mediated inflammasomes, suggesting its role as a potential therapeutic target in IBI.

## 1. Introduction

Stroke is primarily classified into hemorrhagic and ischemic subtypes. Although less fatal, ischemia accounts for 60-80% of all types of stroke globally, significantly impacting human health and quality of life [1]. Ischemic stroke has been correlated with high morbidity rates, physical disability, mortality, and recurrence [2]. Pathologically, ischemia is characterized by cerebral thrombosis or embolism, leading to hypoxia and glucose deficiency. Importantly, sudden depletion of oxygen and glucose most likely induces neuroinflammation and cell death as well as secondary injury to the brain during cerebral ischemia/reperfusion (I/R) and hypoperfusion [3]. A growing body of evidence has revealed the involvement of multimodal pathophysiologies in the cerebral I/R process, resulting in diverse outcomes, such as energy depletion, oxidative stress, excitotoxicity, ion imbalance, and altered gene expression pattern-mediated brain damage and motor/cognitive dysfunctions [4]. Ischemic brain injury (IBI) is unique in that once ischemia begins, irreparable brain damage can occur within minutes to hours [5]. It has been observed that disruption of the blood-brain barrier (BBB) integrity is one of the frequently occurring I/R-induced brain encephalopathy [6]. With the recent advancements in stroke management and rehabilitation, remote ischemic preconditioning (RIPC) therapy has been proven safe and effective in suppressing recurring IS events [7]. However, the main obstacle to IBI treatment remains the efficient and targeted delivery of therapeutics to ischemic foci [8]. Recently, accumulating evidence has pointed out that extracellular vesicles (EVs) possess the potential to treat I/R-induced brain injury in premature infants [9].

EVs are nanosized vesicular structures with lipid bilayer membranes that are secreted into the extracellular space by most cell types under certain pathophysiological conditions [10]. EVs, released by both prokaryotes and eukaryotes, serve as the key mediator of intra- and intercellular communications through physical interactions with the target cell type [11]. Specifically, choroid plexus epithelial (CPE) cell-derived EVs have been identified as novel factors in the signaling communications across the BBB during peripheral inflammation. Additionally, CPE-derived EVs can secrete proinflammatory microRNAs (miRNAs) in the presence of recipient brain parenchymal cells [12]. In particular, miRNA-155 (miR-155) expression has been linked to the promotion of autophagic and neuroinflammatory responses in the IBI [13, 14]. Furthermore, an increased level of matured miR-155-5p in relation to ischemic injury has been validated in the rodent model of middle cerebral artery occlusion (MCAO) as well as in the cell model of induced oxygen and glucose deprivation and subsequent reoxygenation (OGD/R) [15, 16]. Through the bioinformatics analysis of miRNA expression datasets in combination with luciferase reporter assay for miR-155 expression, we found that both miR-155-5p and GTP-binding Ras homolog enriched in brain (Rheb) protein were enriched following the IBI. Rheb has been shown to interact with a myriad of signaling molecules, playing an essential role in regulating apoptosis and autophagy [17, 18]. Rheb also functions as the inducer of mammalian target of rapamycin (mTOR) signaling. It has been found that Rheb/mTOR signaling interaction can inhibit both autophagic and apoptotic responses in neuronal cells, reflecting the induction of underlying neuroprotective mechanism [19]. Stroke pathology-induced miR-155 upregulation mechanistically disrupts the Rheb and mTOR pathway interaction promoting autophagy and inflammasome activation [20]. Among the mTOR complexes, namely, mTORC1 and mTORC2, mTORC1 regulates the translation of its downstream effectors by phosphorylation-mediated posttranslational modifications of 4E-binding protein 1 (4E-BP1), in addition to p70 ribosomal S6 protein kinases (S6Ks) like initiation factors, that modulate cellular proliferation and maturation processes [21].

Rheb expression has been linked to the inhibition of inflammasome activation, particularly the NOD-like receptor family protein pyrin domain-containing 3- (NLRP3-) related inflammasome [21]. The NLRP3 inflammasome signaling modulates cell damage mechanisms by assembling NLRP3 and oligomerized ASC and activates the caspase1 signaling axis in response to microbial infection and other stressors [22]. Suppression of NLRP3 inflammasome activation alleviates IBI pathology [23]. Therefore, we aimed to investigate the mechanism of EV-miR-155-5p-mediated regulation of the Rheb/mTORC1 signaling axis in IBI pathology.

## 2. Materials and Methods

### 2.1. Ethical Approval

The study protocols for animal experiments were approved by the Animals Ethics Committee of the Affiliated Hospital of Guizhou Medical University.

### 2.2. Choroid Plexus Epithelial (CPE) Cell Culture

Mouse primary CPE cells were cultured as described elsewhere [12]. Briefly, 500 mg/kg bodyweight of tribromoethanol was intraperitoneally injected in C57BL/6J mice, aged 2–9 days (provided by Liaoning Changsheng Biotechnology Co. Ltd., Chinese Academy of Sciences, Liaoning, China), for whole body anesthesia, followed by cervical dislocation prior to harvesting the brains. The choroid plexus (CP) was separated under an anatomical microscope. Next, the CP was incubated with streptomycin protease (isolated from *Streptomyces griseus*; Sigma–Aldrich, USA) for 5–7 min for enzymatic hydrolysis, then excess enzyme was removed by washing 2 times with HBSS (Hank's balanced salt solution) medium to terminate the digestion and remove residual enzyme. Cells were then grown in the conditioned medium (CM; DMEM-F12+10% EV-depleted serum) for 48 h on a laminin-coated plate or transwell system. To eliminate the fibroblast cells from the culture, cytosine arabinoside (Ara-C) treatment was initiated after 48 h of culture. Cells were maintained in a humidified chamber at 37°C with 5% CO_2_.

### 2.3. Primary Neuron Culture

Primary cortical neurons were isolated from the cerebral cortex of C57 mouse embryos aged E16 to E18 days. Briefly, cortical neurons (7 × 10^5^ cells/well) were seeded on the poly-D-lysine (Sigma-Aldrich) coated 6-well plate in DMEM medium, which was replaced with B27-supplemented neurobasal medium (Gibco, USA) 4 h postseeding, and was maintained in a moisturized condition with 5% CO_2_ supply for 7-10 days at 37°C, before the experiments.

### 2.4. Construction of an OGD/R Cell Model

CPE cells and primary neurons were washed 2 times with glucose-free DMEM basal medium prior to seeding them for culture in DMEM with 10% FBS in a low-oxygen incubator supplied with 94% N_2_, 5% CO_2_, and 1% O_2_ and then treated with OGD. The culture was removed from the incubator after 45 min for the replacement of the OGD induction medium with the maintenance medium, and the OGD-induction solution was changed with the maintenance medium. Following that, cells were transferred to a conventional cell culture-grade incubator allowing the cells to recover for 24 h for subsequent analyses [24].

### 2.5. Evaluation of EVs

The OGD/R-CPE cell-derived exosomes were isolated in high purity from the supernatant culture medium. Stepwise, after removing the OGD/R-CPE cell culture medium, cells were cleaned on-plate for 2 times with DPBS, and the exosome-free medium (ultracentrifugation at 100,000 × *g* for 16 h at 4°C) was added to the cells to culture for 48 h, prior to collecting the supernatant. The supernatant was then sequentially centrifuged at was at 2,000 × *g* for 30 min and 100,00 × *g* for 30 min and washed for one time in PBS at 100,000 × *g* for 70 min at 4°C. At the final step, exosomes were resuspended for downstream characterizations.

After precipitation, EVs were quickly cross-linked by 2.5% glutaraldehyde (GTA) at 4°C. Then, EVs were subjected to gradient alcohol dehydration steps and finally immersed in epoxy resin. EVs were observed under transmission electron microscopy (TEM) (JEOL 1230, Japan) by staining the finely sectioned slices with lead citrate-uranyl acetate solutions. The remaining portions of the purified EVs were resuspended in PBS buffer to reach the concentration of 10^6^-10^9^ particles/mL for injection into the NanoSight analyzer (ZetaView PMX 110, Germany) using a 1 mL syringe. EV-specific markers, like calnexin, CD63, CD81, and Hsp70, were subsequently analyzed by immunoblotting.

### 2.6. Primary Neuron and EV Coculture

The PKH26 kit (Sigma-Aldrich) was utilized for the coculture as per the manufacturer's manual; EVs were incubated with 2 *μ*M of PKH26 dye (1 : 200) for 5 min. The excess amount of unlabeled dye was removed by passing the suspension through a 100 kDa filter (Microcon, YM-100) 5 times. First, neurons (5 × 10^5^ cells/well) were plated on the 6-well plates in the neuronal basal medium. Then, fluorescent-tagged CPE-derived EVs were added to the neuronal culture at 100 *μ*g/mL concentration and continued the culture in serum-free medium for 12 h prior to induction. Images were captured by Zeiss LSM 780 (Zeiss, Germany) confocal microscope after cells were cultured for 3 days.

### 2.7. Immunofluorescence (IF) Assay

IF was used to identify exosomes absorbed by neurons. The neurons were first washed in chilled PBS, cross-linked by 4% paraformaldehyde (PFA) at room temperature (RT) for 15 min, and treated with 5% serum-containing blocking solution at 37°C for 60 min. After that, cells were probed with anti-MAP2 antibody (#8707; 1 : 200; Cell Signaling Tech (CST)) overnight (ON) at 4°C. The next day, the primary antibody solution was removed, and PBS wash was applied three times before incubating with respective secondary antibody (5529; 1 : 100; CST) at 37°C for 2 h, followed by counterstaining of the nuclei with DAPI for 2 min at 37°C. Finally, the sample was subjected to a one-time PBS wash. IF images were captured by the Olympus confocal microscope (Japan).

### 2.8. Lentiviral Infection

The Rheb coding sequence (GeneChem Co. Ltd., China) was inserted into a self-inactivating pSicoR vector. After annealing, the synthesized oligonucleotide was inserted into the restriction sites of Hpa I and Xho I clones, and the positive clones were identified by restriction enzyme digestion and DNA sequencing. The lentiviral pSicoR vector was cotransfected with psPAX2 and pMD2.G, packaging vectors, in 293T cells. Between 24 h and 72 h postinfection, viral particles were collected by passing the supernatant through a 0.45 *μ*m filter (made of cellulose acetate) every 12 h to obtain the final titer of 109 Tu/mL. Twenty-four-hour preinfection, trypsinized (0.25%) singlet cells (2 × 10^5^ cells/well) were added in 6-well plates containing 10% FBS-supplemented DMEM medium (A2720801; Gibco, USA) without EVs and cultured under standard condition. When the neurons were becoming 30-50% confluent, the nutrient solution was aspirated from the culture. Next, 1 mL/well of a complete nutrient solution containing a 10-fold volume of diluted virus (dilution factor was between 10^−3^ and 10^−7^) was simultaneously added with polybrene (H8761; Solarbio), and these treated neurons were maintained under standard culture condition. The next day, the viral particle-loaded medium was replaced with 2 mL of complete medium for another ON culture. The GFP expression was observed after 5 days with a fluorescence microscope. >95% of cells were found to be GFP-positive. Then, stable positive cells were selected by treating with 0.5 *μ*g/mL of puromycin.

### 2.9. Cell Transfection

CPE cells and neurons were plated separately in 6-well plates at 1 × 10^5^ cells/mL density, one day pretransfection. After reaching between 50% and 70% confluency, successfully modeled CPE cells or neurons were treated with 100 nM of mimic-NC, miR-155-5p mimic, inhibitor-NC, or miR-155-5p inhibitor (GenePharma, China) each by Lipofectamine-2000 (11668027; Thermo Fisher, USA). After modeling, the neurons were immediately transduced with overexpressing- (oe-) NC or oe-NLRP3 adenoviral particles. Cells were selected by G418 (600 mg/L) after 24 h, and the solution was renewed every 3 days. Twelve days postselection, 46% of transduced cells survived. Subsequently, the culture was expanded under a 300 mg/L maintenance concentration to obtain a stably transfected cell line.

### 2.10. Dual-Luciferase Reporter Assay

The synthetic Rheb 3′ UTR (3′ untranslated region) segment was inserted in pmirGLO (Promega, USA) vector by directional cloning. Complementary mutation sites (MUTs) were created by templating the wild-type (WT) Rheb sequence, then target fragments were put in a pgl3 plasmid using DNA T4 ligase following restriction digestion. Then, sequence of MUT luciferase reporter plasmids were verified and then coinserted in HEK-293T (CAS) cells along with miR-155-5p mimic. Then, the luciferase luminescent signal was detected using a luminometer TD-20/20 (E5311; Promega).

### 2.11. Cell Counting Kit-8 (CCK-8)

Cells (5 × 10^4^ cells/mL) were resuspended in DMEM containing 10% FBS. Next, 100 *μ*L of media with cells was put in each well in a 96-well plate and incubated for 24 h, 48 h, and 72 h. After incubation, the supernatant was removed, followed by the addition of 10 *μ*L of CCK-8 solution (WH1199; Shanghai Weiao, China) to every well, and kept for 2 h at 37°C. The absorbance was measured by a microplate reader Multiskan FC (51119080; Thermo Fisher) [24]. Triplicated wells were measured for each group to calculate the mean value.

### 2.12. TUNEL Staining

We performed the TUNEL assay to determine whether EV treatment reduced the apoptosis rate of cortical neurons. The cells were treated with 4% PFA. Following PBS washing, a TUNEL assay was performed using fluorescein-coupled probes, as directed in the protocol. Cells were then counterstained with DAPI (1 : 1000; Beyotime, China) for 10 min at room temperature to evaluate nuclear morphology. Each slide was washed, and images were captured by a fluorescence microscope. To assess whether the EV treatment reduced the neuronal apoptosis rate, we performed the TUNEL assay. We calculated the populations of TUNEL+NeuN and NeuN-only positive cells in 5 distinct brain regions. In this *in vitro* study, we calculated the number of TUNEL+DAPI double-positive cells and only DAPI-positive cells in the similar 5 brain regions in each slide for statistical analysis. The ratio of injured to uninjured cells was calculated by dividing the count of DAPI/TUNEL double-positive cells to only DAPI-positive cells.

### 2.13. Construction of a Transient Middle Cerebral Artery Occlusion (tMCAO) Mouse Model

All adult male C57BL/6J mice were 8–10 weeks old (provided by Liaoning Changsheng Biotechnology Co. Ltd., China). At all stages of the study, the researchers were unaware of the experimental conditions. Mice were randomly assigned to different treatment groups: (1) sham group: mice that underwent sham surgery; (2) model group: no intervention; (3) model intervention group: NC EV-agomir, EV-miR-155a-5p agomir, NC antagomir, EV-miR-155a-5p antagomir, oe-NC, oe-Rheb, oe-Rheb+EV-agomir-NC, and oe-Rheb+EV-miR-155a-5p agomir-treated groups. They were given *ad libitum* access to fine-grained feed and water and provided with natural light in a 12 h light/dark cycle. After anesthetizing mice with 30–70% oxygen/nitrous oxide combined with 1.5–2% isoflurane, a 6-0 nylon suture (silicone-coated) was guided through the external to the internal carotid artery and then to the MCA. The efficiency of the occlusion model was determined from the surface cerebral blood flow (CBF) rate by a laser Doppler flowmeter (Moor Instruments, UK) until 10% of the baseline CBF was reached. One hour after successful occlusion, the suture was taken out to allow reperfusion, and immediately after this, EVs were administered through the tail vein at 100 *μ*g per day dose for 3 days [25].

### 2.14. Intracerebral Injection

miR-155-5p agomir, NC agomir, miR-101a-3p antagomir, and NC antagomir (2.5 *μ*g/2.5 *μ*l each), purchased from GenePharma (Shanghai, China), were mixed with 1.25 *μ*l of EntransterTM *in vivo* transfection reagent (18668-11-1l; Engreen, China). Then, 1.25 *μ*l of PBS was added to the mixture and kept for 5 min at room temperature before intracerebroventricular (i.c.v.) injection using a microsyringe (ZS Dichuang Co., China) and a stereotaxic instrument (ZS Dichuang Co., China). Three days after tMCAO, mice were euthanized to harvest brain samples, which were immediately kept in a freezing mold with antifreeze (Thermo Scientific™) to be stored at -80°C.

### 2.15. Neurobehavioral Tests

Neurobehavioral examinations were conducted before and after 3 days of the establishment of the tMCAO model by investigators unaware of the experimental design. The neurological assessment was based on the modified neurological severity score (mNSS) system, which combines reflex, motor, and balance test scores [26]. The severity score ranges from 0 to 14, where 0 indicates normal, and increasingly higher scores indicate increasing injury severity [27].

### 2.16. Brain Weight

Three days after tMCAO surgery, mice were euthanized, and the brains were collected without perfusion. After dissecting out the cerebellum and brainstem portions from the forebrain, the remaining whole brain was cut through the midline and weighed using a precision balance (sensitivity, 0.001 g). Then, the mass ratio of the ipsilateral (right) to the contralateral (left) hemisphere was calculated.

### 2.17. Triphenyltetrazolium Chloride (TTC) Treatment

The whole brains were harvested without perfusion from the EV-treated tMCAO mice after proper euthanasia. The brain was sliced into 2 mm sections, incubated in TTC solution (2%) (Sigma-Aldrich) in the dark for 10 min, and fixed with 10% PFA. The cerebral infarction volumes were analyzed with ImageJ (NIH, USA). The percentage of infarcts was determined using the following formula: (total area of the contralateral hemisphere − no infarction area of the ipsilateral hemisphere)/(total area of the contralateral hemisphere × 2).

### 2.18. Enzyme-Linked Immunosorbent Assay (ELISA)

ELISA kits (69-21178 and 69-21183; MSKBIO, China) were used to determine the levels of IL-18 and IL-1*β* in the mouse brain tissue lysates. Mouse brain tissues in sterile PBS were ground, the mixture was centrifuged at 10000 rpm for 10 min, and the supernatant was collected for detection. The absorbance (A) values of the wells were measured at 450 nm within 3 min of preparation using an all-purpose enzyme marker (Synergy 2, BioTek, Winooski, VT, USA).

### 2.19. Real-Time Quantitative-Polymerase Chain Reaction (RT-qPCR)

EV-containing miRNAs were purified using the SeraMir exosome RNA purification kit (System Biosciences, USA). Total cellular RNA was extracted by the TRIzol method (15596026; Invitrogen). Complementary DNA (cDNA) was prepared from miRNA by miScript Reverse Transcription kit (Qiagen GmbH, Germany). U6 served as the internal control in the stem-loop RT-qPCR assay (GenePharma, China).

A PrimeScript™ RT reagent kit (TaKaRa) was employed for cDNA synthesis from 1 *μ*g of total RNA. Next, a StepOnePlus™ RT-PCR System (Invitrogen) was utilized for quantitative mRNA expression analysis of *Rheb*, *NLRP3*, and transthyretin (*TTR*) genes. The primer sequences are listed in Table 1. *β*-Actin served as an internal reference. SYBR® Premix Ex Taq™ (Tli RNaseH Plus) (TaKaRa) was used for cDNA amplification. The 2^-*ΔΔ*Ct^ method was used to calculate the relative mRNA or miRNA expressions.

### 2.20. Western Blot Analysis

Cells or tissue lysates were prepared by lysing the sample in lysis buffer (1 mL) with protease inhibitor (P0013J; Beyotime) for 45 min on ice, followed by spinning at 4000 × *g* for 30 min at 4°C. The protein concentration of each supernatant sample was measured using the BCA kit (PC0020; Solarbio). The 20 *μ*g protein sample from each group was resolved by sodium dodecyl sulfate-polyacrylamide gel electrophoresis (SDS-PAGE). Next, proteins were transferred to a polyvinylidene fluoride (PVDF) membrane (66485; Pall Corp, USA). The membrane was then blocked with 5% nonfat skim milk for 2 h at RT. The membranes were then probed with respective primary antibodies, anti-Rheb (ab36988; 1 : 1000; Abcam, UK), anti-mTOR (ab36988; 1 : 2000; Abcam, UK), anti-4EBP1 (ab36988; 1 : 2000; Abcam, UK), anti-S6K1 (ab36988; 1 : 5000; Abcam, UK), anti-Beclin-1 (ab207612; 1 : 2000; Abcam), anti-LC3A/B (to detect LC3 II/I; 12741; 1 : 1000; CST), anti-LAMP-1 (ab108597; 1 : 1000; Abcam), anti-CD63 (25682-1-AP; 1 : 1000; Proteintech, USA), anti-P62 (ab155686; 1 : 3000; Abcam), anti-NLRP3 (15101; 1 : 1000; CST), anti-CD81 (ab109201; 1 : 1000; Abcam), anticalnexin (ab10286; 1 : 2000; Abcam), anticleaved-caspase3 (9664; 1 : 1000; CST), anticaspase3 (9662; 1 : 1000; CST), antiheat shock protein 70 (Hsp70) (ab181606; 1 : 1000; Abcam), and anti-*β*-actin (ab8227; 1 : 1000; Abcam) overnight at 4°C. Then, corresponding horseradish peroxidase- (HRP-) linked IgG (ab6721; 1 : 2000; Abcam) secondary antibodies were probed for 1 h at RT. The membrane was treated in electrogenerated chemiluminescence (ECL) solution (BM101; Biomiga, USA) for 1 min at room temperature. The blots were observed in the dark using a chemiluminescence analyzer with *β*-actin as the loading reference.

### 2.21. Statistical Analysis

SPSS 21.0 software (IBM, USA) was used for the statistical analyses. The data were expressed as means ± standard deviation (SD). An unpaired *t* test was conducted for comparisons of data between two groups. For multiple group comparisons, a one-way analysis of variance (ANOVA) was performed, followed by Tukey's post hoc test. For comparisons of data between groups at different time points, two-way ANOVA was performed, followed by Bonferroni's test. Statistical significance was established at *p* < 0.05.

## 3. Results

### 3.1. CPE Cell-Derived EVs Promote Neuronal Injury

**Figure 1:**
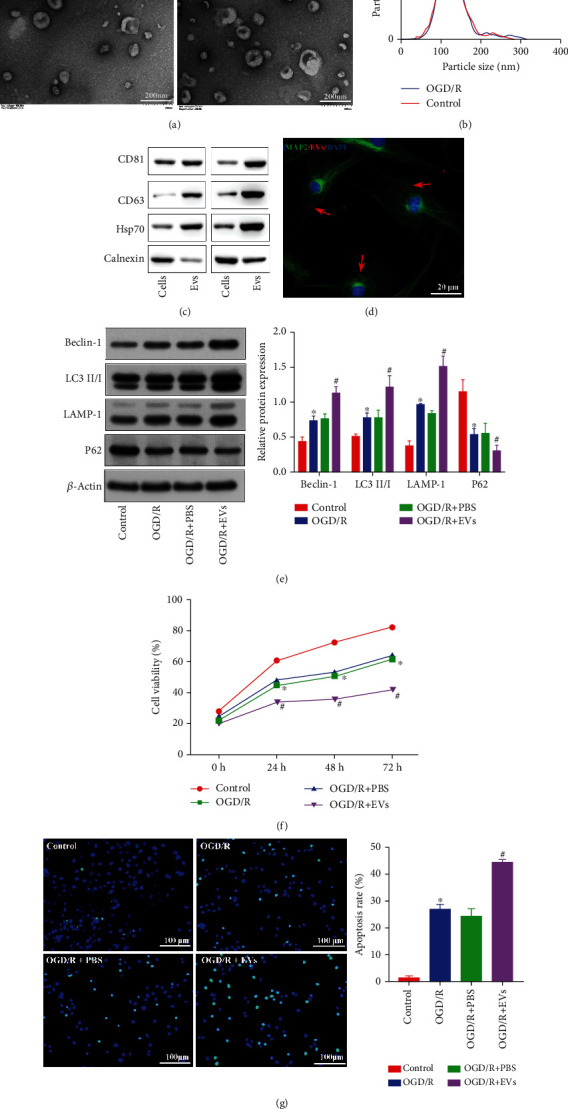
CPE-derived EVs promote autophagy and apoptosis. (a) EV morphology was observed by TEM. (b) NanoSight NTA (nanoparticle tracking analysis) of the EVs. (c) EV marker protein expression was measured by immunoblotting. (d) The interaction of fluorescent-labeled EVs and neurons under OGD/R conditions was detected by laser confocal microscopy. (e) Autophagy-related protein expressions in the cell model after coculture was determined by immunoblotting. (f) The viability of neurons exposed to OGD/R after coculture was determined using a CCK-8 kit. (g) The number of TUNEL^+^ neurons exposed to OGD/R after coculture was determined by TUNEL staining. ^∗^*p* < 0.05 vs. the brain group or control group. ^#^*p* < 0.05 vs. neurons treated with OGD/R+PBS (*n* = 3).

To investigate the regulatory mechanisms of CPE cell-derived EVs in IBI, we established a mouse primary CPE cell model. Next, the CPE cells were subjected to an OGD/R environment, and EVs were subsequently isolated from the normal and OGD/R culture. We observed the typical cup-shaped EV morphology under TEM. Membranous structures were visualized around the vesicles with the central low electron density region (Figure 1(a)). The NanoSight tracking analysis revealed that the EVs had a diameter between 40 and 160 nm exhibiting irregular Brownian motion, and the number of CPE cell-derived EVs was increased when exposed to OGD/R (Figure 1(b)). Western blot analysis indicated that CPE-derived EVs could express the relevant marker proteins CD81, CD63, and Hsp70 but not the calnexin (Figure 1(c)), suggesting that the ischemic environment stimulated the production of more EVs in CPE cells. Therefore, subsequent experiments were conducted using EVs isolated from OGD/R-treated CPE cells.

To verify whether CPE-derived EVs could enter neurons and affect their functions, we first constructed an OGD/R model with neurons. Subsequently, PKH26-labeled EVs were cocultured with the neuron for 6 h, and CPE-derived EVs were found to be absorbed by neurons (Figure 1(d)). Next, to examine the effect of these EVs on neurons, we detected the formation of autophagosomes in neurons and measured the expression of related proteins (Figure 1(e)), assessed cell viability (Figure 1(f)), and determined the apoptosis rate (Figure 1(g)). The number of autophagosomes was significantly increased, and LC3 II/I, Beclin-1, and LAMP-1 expressions were notably elevated, but P62 expression was strikingly downregulated (Figure 1(e)), with decreased cell viability (Figure 1(f)), and increased the number of TUNEL-positive (Figure 1(g)) neurons exposed to OGD/R compared with normal controls. Additionally, neurons cotreated with OGD/R+EV showed an increased number of autophagosomes; elevated Beclin-1, LC3 II/I, and LAMP-1 protein expression; decreased P62 protein expression (Figure 1(e)); decreased cell viability (Figure 1(f)); and an increased number of TUNEL-positive cells (Figure 1(g)) compared with neurons cotreated with OGD/R+PBS. These results suggest that CPE-derived EVs can promote neuronal autophagy and apoptosis.

### 3.2. CPE Cell-Derived EVs Aggravate Neuronal Injury through miR-155-5p

**Figure 2:**
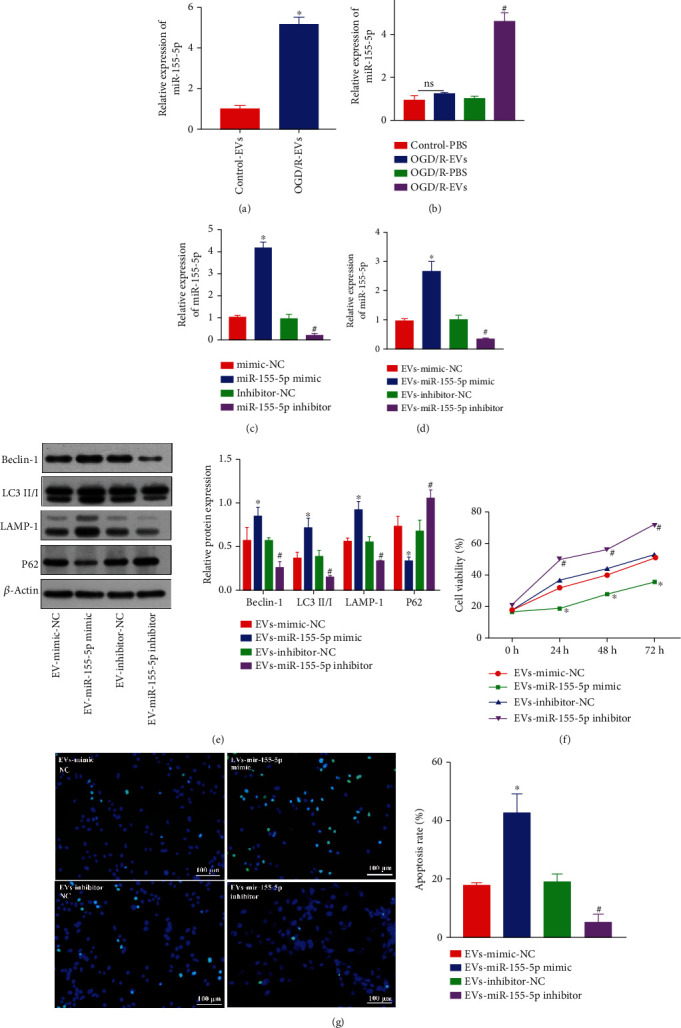
CPE-derived EVs deliver miR-155-5p to regulate neuronal injury. (a) The expression of miR-155-5p in CPE-derived EVs was measured by RT-qPCR. (b) The expressions of miR-155-5p in cocultured cell models after adding EVs were examined using RT-qPCR. (c) The transfection efficiency of miR-155-5p in CPE cells was quantified by RT-qPCR. (d) The expression of miR-155-5p in a cocultured cell model was determined by RT-qPCR. (e) The expression of autophagy-related proteins in the cell model after coculture was measured by western blot analysis. (f) The viability of neurons was measured by CCK-8 assay. (g) The number of TUNEL^+^ neurons was determined by TUNEL staining. ^∗^*p* < 0.05 vs. mimic-NC-transfected neurons, control- and EV-cotreated neurons, or EV- and mimic-NC-cotreated neurons. ^#^*p* < 0.05 vs. inhibitor-NC-transfected neurons, OGD/R-PBS-treated neurons, or EV- and inhibitor-NC-cotreated neurons (*n* = 3).

CPE-derived EVs can secrete proinflammatory miRNAs that affect recipient brain parenchymal cells [12]. Furthermore, miR-155 promotes autophagy [13]. Therefore, we hypothesized that CPE-derived EVs might regulate autophagy in neurons by secreting miR-155, thereby alleviating IBI. Hence, miR-155-5p expression in EVs was determined using RT-qPCR, which showed significantly increased expression of miR-155-5p when cells were exposed to OGD/R (Figure 2(a)), indicating that hypoxia can induce CPE cell-derived EVs to produce miR-155-5p.

Next, EVs were first added to neuron culture exposed to OGD/R condition, and then, the miR-155-5p level was determined. Compared with that in cultured EV-treated neurons under normal conditions, miR-155-5p was several-fold increased in EV-treated neurons under the OGD/R condition (Figure 2(b)). CPE cells were then transfected with the miR-155-5p mimic and miR-155-5p inhibitor (Figure 2(c)). To verify that neurons can be transfected with miR-155-5p mimic/inhibitor, neurons were then transfected with the miR-155-5p mimic and miR-155-5p inhibitor (Figure S1). EVs and neurons were isolated and cocultured under the OGD/R condition. miR-155-5p expression in neurons was measured by RT-qPCR, revealing that miR-155-5p expression in neurons cotreated with EVs and miR-155-5p mimic was significantly elevated compared with that in neurons cotreated with EVs and mimic-NC. Additionally, miR-155-5p expressions in EV- and miR-155-5p inhibitor-cotreated neurons were notably decreased compared with that in EV- and inhibitor-NC-cotreated neurons (Figure 2(d)). Furthermore, compared with that in EV- and mimic-NC-cotreated neurons, neurons cotreated with EVs and miR-155-5p mimic could induce increased autophagosome formation, elevated expressions of LC3 II/I, Beclin-1, and LAMP-1 proteins, decreased P62 expression (Figure 2(e)), reduced cellular activity (Figure 2(f)), and increased TUNEL-positive cell count (Figure 2(g)). An opposite trend was observed following the treatment of miR-155-5p inhibitor.

### 3.3. CPE Cell-Derived EVs Aggravate IBI through miR-155-5p

**Figure 3:**
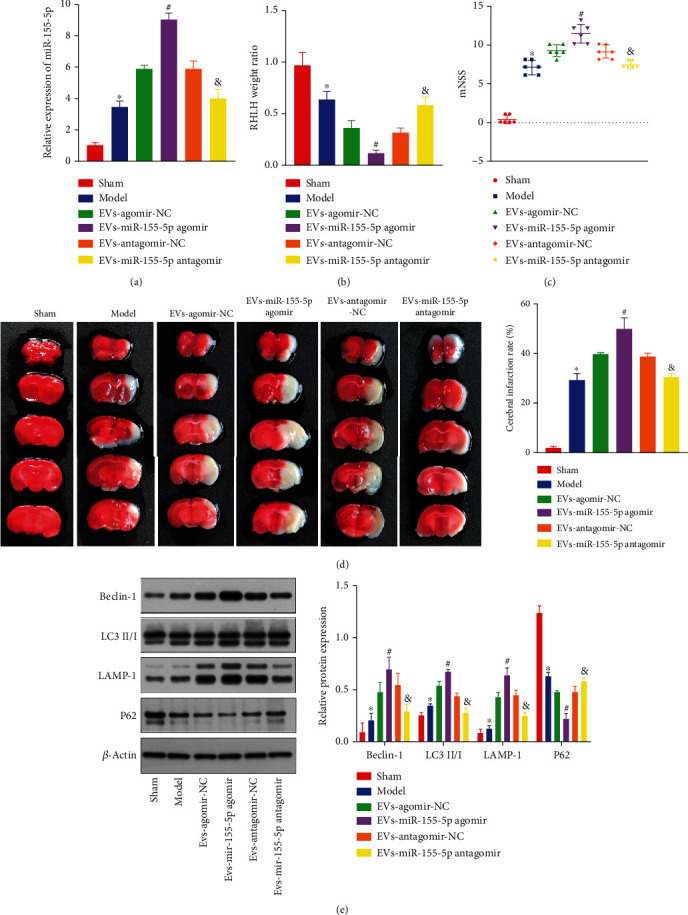
CPE-derived EVs deliver miR-155-5p to aggravate brain injury in mice. (a) miR-155-5p expressions in mouse brain tissues were estimated using RT-qPCR. (b) The mass ratio of the ipsilateral (right) hemisphere to the contralateral (left) hemisphere of the brain in tMCAO model mice was calculated. (c) The neurological function of mice was determined by mNSS. (d) Cerebral infarctions in tMCAO model mice were detected by TTC. (e) Immunoblotting for autophagy-related protein levels in mouse brain tissues. ^∗^*p* < 0.05 vs. sham-operated mice. ^#^*p* < 0.05 vs. EV- and agomir-NC-cotreated mice. ^&^*p* < 0.05 vs. mice cotreated with EVs and antagomir-NC (*n* = 3).

Next, a tMCAO mouse model was constructed and administered with miR-155-5p mimic or inhibitor treated CPE-derived EVs. Compared with the mice receiving sham treatment, the miR-155-5p level was significantly increased (Figure 3(a)), the mass ratio of the ipsilateral (right) hemisphere to the opposite (left) hemisphere of the brain was significantly reduced (Figure 3(b)), the mNSS was increased (Figure 3(c)), the cerebral infarction rate was significantly increased (Figure 3(d)), and the levels of LC3 II/I, Beclin-1, and LAMP-1 were increased considerably, while P62 expression was significantly reduced (Figure 3(e)) in the mice that had undergone the tMCAO operation, suggesting the successful establishment of the tMCAO model. Compared with mice treated with EV+mimic-NC, mice treated with EV+miR-155-5p mimic exhibited a similar trend. However, compared with mice cotreated with EV+inhibitor-NC, mice cotreated with EV+miR-155-5p inhibitor exhibited opposite results. These results indicated that CPE-derived EVs promoted autophagic activity, activated inflammatory factors, and aggravated IBI through miR-155-5p expression in the tMCAO mouse model.

### 3.4. miR-155-5p in CPE Cell-Derived EVs Can Target and Inhibit Rheb to Enhance Neuronal Injury

**Figure 4:**
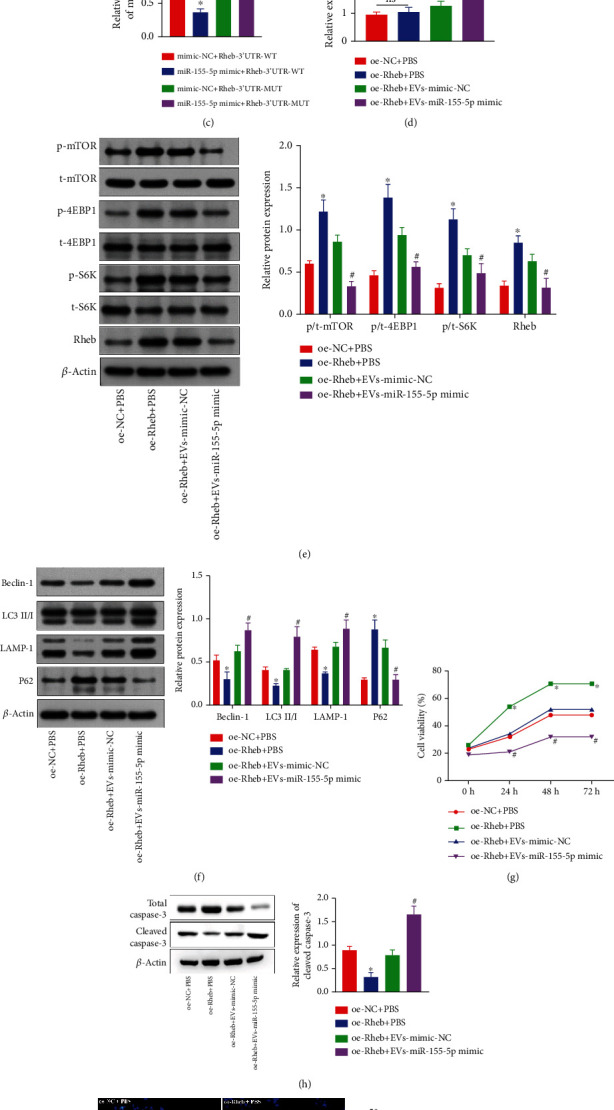
miR-155-5p in CPE-derived EVs inhibits Rheb to promote neuronal autophagy and apoptosis. (a) Target genes of miR-155-5p were predicted by analyzing the miRDB, TargetScan, and RNA22 databases. (b) The binding sites of miR-155-5p and Rheb in humans, rats, and mice were obtained from the TargetScan analysis. (c) Crosstalk between miR-155-5p and Rheb was determined by luciferase assay. (d) Relative miR-155-5p level in cocultured cell models was determined by RT-qPCR. (e) Phosphorylation levels of mTOR, S6, and 4E-BP1 in the cell model after transfection were confirmed by western blotting. (f) Expressions of autophagy-related proteins in the cell model after transfection and coculture were measured by western blotting. (g) Activity of neurons treated with OGD/R after transfection and coculture was measured by CCK-8 assay. (h) Levels of total caspase3 and cleaved-caspase3 proteins in the cell model after coculture were detected by western blotting. (i) The number of TUNEL^+^ neurons exposed to OGD/R after coculture was determined by TUNEL staining. ^∗^*p* < 0.05 vs. mimic-NC+Rheb-3′ UTR-WT cotreated neurons, mimic-NC-transfected neurons, controls, oe-NC-transfected neurons or oe-NC+PBS-cotreated neurons. ^#^*p* < 0.05 vs. neurons cotreated with inhibitor-NC or oe-Rheb+EV and mimic-NC (*n* = 3).

We analyzed the miRDB, TargetScan, and RNA22 databases to predict the targets of miR-155-5p and found that Rheb was the only gene regulated by miR-155-5p identified in each of these datasets (Figure 4(a)). As shown in Figure 4(b), the binding sites of miR-155-5p and Rheb in humans, rats, and mice were identified using TargetScan. Additionally, the Rheb/mTORC1 axis exerts inhibitory effects on autophagy and inflammasome activation following stroke [20]. Therefore, we hypothesized that miR-155-5p might regulate neuronal function *via* the Rheb/mTORC1 axis. The luciferase assay showed that the bioluminescence intensity in neurons cotreated with the miR-155-5p mimic and Rheb-3′ UTR-WT was significantly reduced, as compared with that of mimic-NC plus Rheb-3′ UTR-WT cotransfected cells, suggesting that miR-155-5p could bind to the Rheb gene specifically (Figure 4(c)).

We examined whether miR-155-5p delivered to neurons by CPE-derived EVs could inhibit Rheb/mTORC1 activity and thereby might affect neuronal function. We observed that the miR-155-5p level was drastically elevated, whereas Rheb expression was decreased in oe-Rheb+EV- and miR-155-5p mimic-cocultured neurons compared with that in oe-Rheb+EV- and mimic-NC cotreated cells (Figure 4(d)). Therefore, increased phosphorylations of mTOR, S6K, and 4EBP1 were observed in neurons cotreated with oe-Rheb+PBS compared with neurons cotreated with oe-Rheb+EV- and miR-155-5p-cotreated mimic (Figure 4(e)).

Furthermore, neurons cotreated with oe-Rheb+PBS showed inhibited autophagosome formation; decreased LC3 II/I, Beclin-1, and LAMP-1 protein levels; increased P62 expression; enhanced activity (Figures 4(f) and 4(g)); reduced cleaved-caspase3 protein expression; and fewer TUNEL-positive neurons (Figure 4(h)) than oe-NC+PBS-cotreated neurons, and these effects were abrogated following further treatment with EV- and miR-155-5p mimic cotreatment. In summary, EVs derived from CPE cells delivered miR-155-5p to neurons, suppressing Rheb/mTORC1 activity, promoting neuronal autophagy and apoptosis, and diminishing neuronal viability.

### 3.5. Rheb Activates the NLRP3 Inflammasome to Promote Neuronal Autophagy and Apoptosis

**Figure 5:**
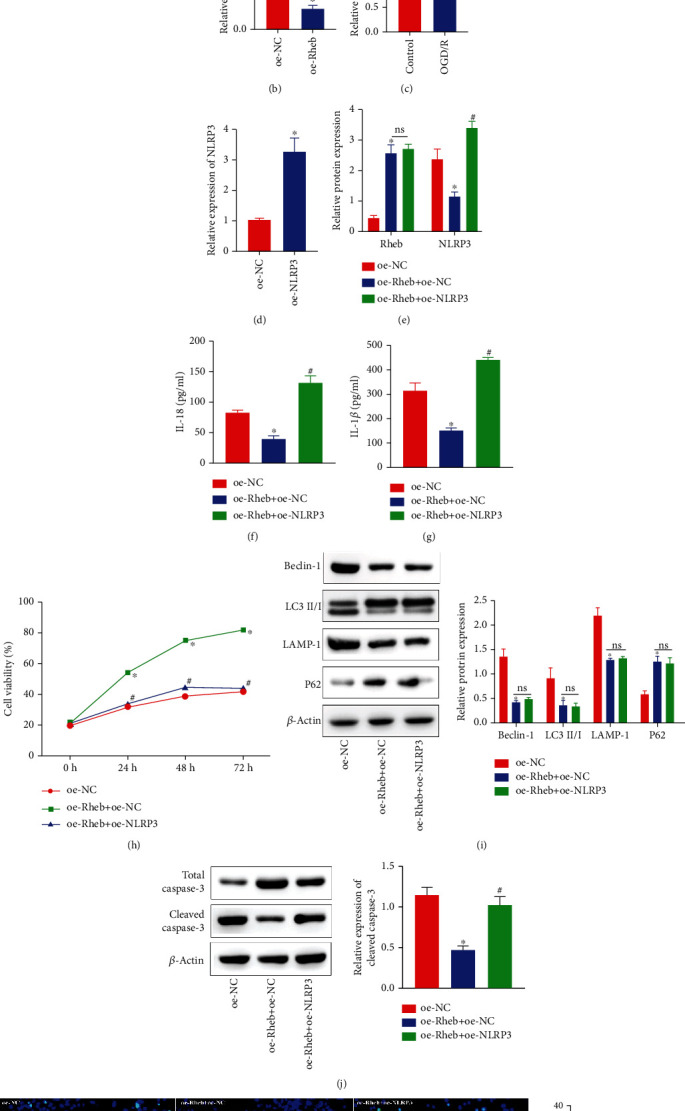
Rheb activates the NLRP3 inflammasome to enhance neuronal autophagy and apoptosis. (a) Rheb and NLRP3 were coexpressed as determined using the MEM tool. (b) NLRP3 expression was estimated by RT-qPCR. (c) NLRP3 expression in the OGD/R-exposed cell model was measured by RT-qPCR. (d) oe-NLRP3 neuron transfection efficiency was quantitated by RT-qPCR. (e) Rheb and NLRP3 expression in oe-Rheb+oe-NC cotransfected neurons exposed to OGD/R conditions as measured by RT-qPCR. (f, g) The secretion levels of IL-18 (f) and IL-1*β* (g) from neurons cotransfected with oe-Rheb+oe-NC and exposed to OGD/R conditions were measured by ELISA. (h) The activity of neurons was determined using a CCK-8 kit. (i) Expressions of autophagy-linked proteins (Beclin-1, LC3 II/I, LAMP-1, and P62) and NLRP3 inflammasome activation-related proteins as measured by western blot analysis. (j) Protein expressions of total caspase3 and cleaved-caspase3 in neurons were determined by western blot analysis. (k) The number of TUNEL^+^ neurons was determined by TUNEL staining. ^∗^*p* < 0.05 vs. control or oe-NC-transfected neurons. ^#^*p* < 0.05 vs. oe-Rheb+oe-NC-cotransfected neurons (*n* = 3).

The NLRP3-mediated activation of the inflammasome is characterized by upregulated NLRP3 expression [28]. Expression analysis of publicly available databases was performed using the MEM tool (https://biit.cs.ut.ee/mem/index.cgi), and Rheb and NLRP3 were found to be coexpressed (Figure 5(a)). NLRP3 expression was reduced when Rheb was overexpressed (Figure 5(b)). Furthermore, NLRP3 expression in OGD/R-exposed neurons was increased compared with that in control neurons (Figure 5(c)). To explore the regulatory connection between Rheb and NLRP3 in neurons, we overexpressed Rheb and NLRP3 in cell models. RT-qPCR showed that NLRP3 expression in oe-NLRP3-transfected neurons was notably elevated (Figure 5(d)). Subsequently, compared with oe-NC-transfected neurons, neurons cotransfected with oe-Rheb+oe-NC exhibited notably increased Rheb expression but downregulated Rheb expression (Figure 5(e)). Rheb overexpression in neurons resulted in reduced proinflammatory factor (IL-18 and IL-1*β*) levels (Figures 5(f) and 5(g)); inhibited autophagosome formation; downregulated Beclin-1, LC3 II/I, and LAMP-1 expressions; elevated P62 level; inhibited NLRP3 inflammasome activation (Figure 5(i)); and enhanced cell viability (Figure 5(h)), thus reducing the cleaved-caspase3 protein level (Figure 5(j)) and fewer TUNEL-positive cells (Figure 5(k)). These effects were abrogated by treatment with oe-NLRP3. Therefore, Rheb can activate the NLRP3 inflammasome to promote neuronal autophagy and apoptosis.

### 3.6. miR-155-5p in EVs Derived from CPE Cells Suppresses Rheb Expression to Aggravate IBI

**Figure 6:**
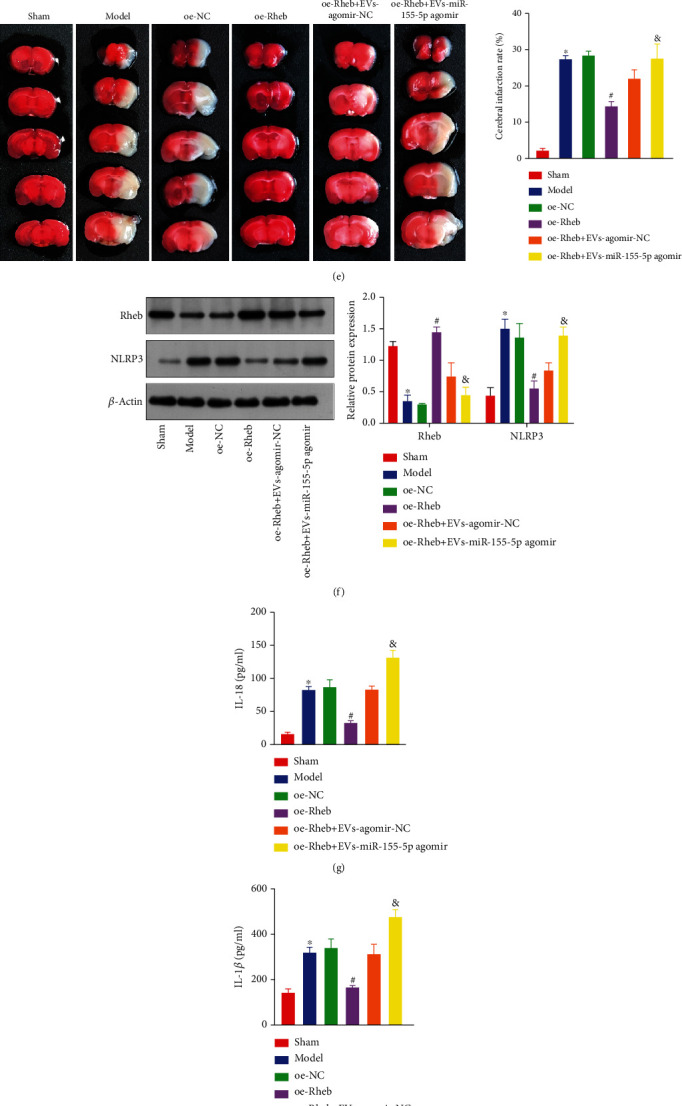
CPE-derived EVs containing miR-155-5p suppressed Rheb to aggravate IBI. (a) The expressions of miR-155-5p in mouse brain tissues were determined by RT-qPCR. (b) Rheb mRNA levels in mouse brains were determined by RT-qPCR. (c) The mass ratio of the ipsilateral (right) hemisphere to the contralateral (left) hemisphere of the brain in tMCAO mice was calculated. (d) The neurological function of mice was measured by mNSS. (e) Cerebral infarction in tMCAO mice was detected by TTC. (f) Protein levels of Rheb, mTORC1, and NLRP3 in brain tissues were detected by western blotting. (g, h) Levels of IL-18 (g) and IL-1*β* (h) in the brain tissue extracts of tMCAO mice were determined by ELISA. (i) Autophagy-associated proteins' expressions in cotreated mouse brain tissues were measured by western blotting. ^∗^*p* < 0.05 vs. sham-operated mice. ^#^*p* < 0.05 vs. oe-NC-infected mice. ^&^*p* < 0.05 vs. mice cotreated with oe-Rheb+EV and agomir-NC (*n* = 3).

To further understand the mechanism of CPE-derived EVs on IBI in tMCAO mice, we treated mice with oe-Rheb+EV with mimic-NC or oe-Rheb+EV with miR-155-5p mimic. RT-qPCR indicated an increase of the miR-155-5p expression with a decreased Rheb expression in the tMCAO model compared with the corresponding expression levels in sham-treated mice. Interestingly, miR-155-5p expression remained unchanged, whereas Rheb expression was elevated in oe-Rheb-infected mice compared with the corresponding expression levels in tMCAO and oe-NC-infected mice, but this trend was reversed by EV and miR-155-5p mimic cotreatment, which also led to increased miR-155-5p expression (Figures 6(a) and 6(b)).

Compared with that in sham-treated mice, the mass ratio of the ipsilateral (right) to the contralateral (left) hemisphere in the mouse model of tMCAO was reduced (Figure 6(c)), and the mNSS (Figure 6(d)) and cerebral infarction rates were increased (Figure 6(e)). The expression was significantly reduced, and NLRP3 expression was sharply increased (Figure 6(f)). The levels of proinflammatory factors IL-18 and IL-1*β* in the mouse brain tissue lysates were remarkably increased (Figures 6(g) and 6(h)). The protein expressions of Beclin-1, LC3II/I, and LAMP-1 in the brain tissues of these mice were significantly increased, and P62 expression was dramatically reduced (Figure 6(i)).

Compared with tMCAO- and oe-NC-infected mice, the mass ratio of the ipsilateral (right) hemisphere to the contralateral (left) brain hemisphere in the oe-Rheb-infected mice was significantly increased, while the mNSS and cerebral infarction rates were significantly reduced. The expression was increased, and NLRP3 expression was significantly reduced. The IL-18 and IL-1*β* concentrations in the brain samples were strikingly decreased. The LC3II/I, Beclin-1, and LAMP-1 expressions in the brain tissues were significantly reduced, but P62 expression was notably increased, the effect of which was rescued by EV and miR-155-5p mimic cotreatment. Notably, miR-155-5p in CPE cell-derived EVs suppressed Rheb to aggravate IBI.

## 4. Discussion

Stroke is characterized as a cerebrovascular disease, leading to high disability and mortality rates, especially among the elderly population [29, 30]. Increasing evidence has highlighted that EVs are critically involved in the ischemic pathomechanism, and EVs derived from different cell types can induce neuroprotection and neurorestorative effects by modulating gene, protein, and miRNA expressions in their target cell and tissue types [31]. However, to date, studies have focused only on the potential of EVs in treating ischemic stroke. Previous studies have revealed that LPS-stimulated primary CPE cell-derived EVs can enter the brain parenchyma to inhibit the miRNA targets and inflammatory gene upregulation, resulting in aggravated brain injury [12]. Studies on the mechanisms involved in brain injury after cerebral ischemia have mainly investigated neuronal responses after ischemic stroke. No study has revealed the molecular signal mediating interactions between CPE cells and neurons to exacerbate a neuronal injury. Identifying the molecular mechanism by which CPE cells contribute to neuronal injury would facilitate the development of new strategies to treat ischemic stroke.

Initially, we found that CPE cell-derived EVs promoted cell inflammation, as evidenced by increased IL-18 and IL-1*β* levels; enhanced autophagy characterized by increased LC3II/I, Beclin-1, and LAMP-1 levels; reduced P62 levels; decreased cell viability; and elevated apoptosis rate. Similar findings have been previously reported; for example, the addition of EVs increased the expression of Beclin-1 and LC3B II/I in mice after focal brain irradiation [32]. Mesenchymal stem cell-derived EVs can inhibit the proliferation/migration by the ERK pathway activation in RSC96 cells, promoting apoptosis [33]. IL-18 acts as the proinflammatory cytokine inducing tissue damage, inflammation, and apoptosis, and increased IL-18 levels are associated with myocardial injury after ischemia or infarction [34]. Our experiments revealed that CPE-derived EVs could deliver miR-155-5p to aggravate neuronal injury. Therefore, CPE cell-derived EVs constituted a novel mechanism of communication between the peripheral blood under inflammatory conditions and the brain, with systemic inflammation resulting in an increase in EVs and associated proinflammatory miRNAs, including miR-155 [12]. It has been observed that the miR-155-5p level is increased in EVs present in the cerebrospinal fluid [35]. Additionally, a previous study showed that miR-155-5p restoration could promote autophagy, while miR-155-5p inhibition could decrease P62 expression [36].

Subsequently, our study findings suggested that miR-155-5p expression could impact the IBI through Rheb signaling. Reportedly, miR-155 binds the 3′ UTR of Rheb and inhibits its expression [20]. The same report suggests that reduced Rheb expression downregulates mTORC1 expression, which is associated with considerable cerebral infarction volumes and cell apoptosis during ischemic stroke [20, 37]. Consistently, we found that CPE cell-derived EVs contained miR-155-5p that promoted neuronal injury by targeted inhibition of Rheb expression. Earlier, it was shown that Rheb activation promotes the differentiation of neurons [38]. Rheb also inhibits NLRP3 inflammasome activation [21]. Additionally, NLRP3 is overexpressed in cells exposed to OGD/R conditions, whereas NLRP3 inhibitor treatment decreases NLRP3 expression in ischemic stroke [39]. Furthermore, studies have revealed that inhibition of NLRP3 inflammasome activation can attenuate hypoxic IBI in newborn male rats [23]. NLRP3 inhibitors clearly have unique anti-inflammatory effects, protecting the injured brain after traumatic brain injury [40].

## 5. Conclusion

**Figure 7:**
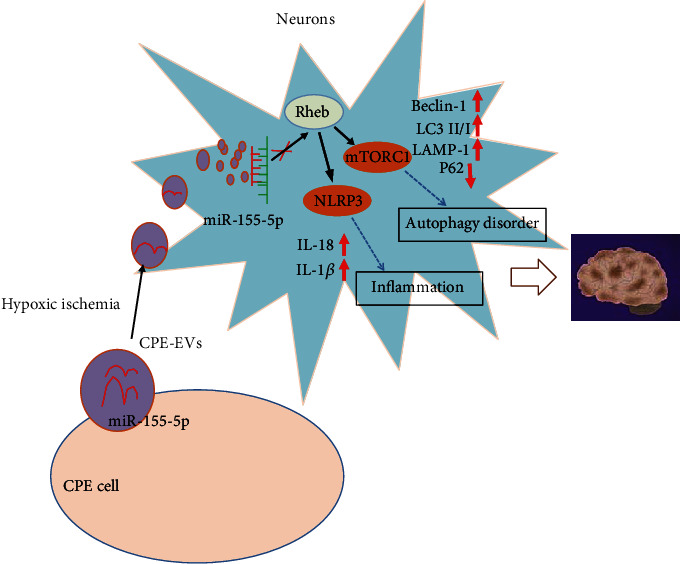
Schematics of potential mechanisms involved in the EV-delivered miR-155-5p and IBI. CPE-derived EV-derived miR-155-5p promoted inflammation- and/or autophagy-related protein expression to promote inflammation and autophagy of neurons through the Rheb/mTORC1-NLRP3 axis in a hypoxic environment.

In summary, CPE cell-derived EVs containing miR-155-5p can aggravate IBI by suppressing the Rheb/mTORC1 expression and activating the NLRP3-mediated inflammasome, highlighting the miR-155-5p expression as the potential therapeutic target in IBI (Figure 7). Progressive steps to elucidate the mechanisms of IBI and prevent its incidence are urgently needed in the future.

## Figures and Tables

**Table 1:** RT-qPCR primers.

Target	Forward primer (5′-3′**)**	Reverse primer (5′-3′**)**
miR-155-5p	TTAATGCTAATCGTGATAGGGGT	GCTGTCAACGATACGCTACGTAACG
Rheb	AGGAAAGTCTTCGTGCTCGG	GAGGACCCCTGACCCAAATG
NLRP3	ATTACCCGCCCGAGAAAGG	CATGAGTGTGGCTAGATCCAAG
U6	ATGGGTCGAAGTCGTAGCC	TTCTCGGCGTCTTCTTTCTCG
*β*-Actin	TGTTACCAACTGGGACGACA	GGGGTGTTGAAGGTCTCAAA

Note: miR-155-5p: microRNA-155-5p; Rheb: Ras homolog enriched in brain; NLRP3: NOD-like receptor protein 3; U6: U6 small nuclear RNA (snRNA); *β*-actin: beta-actin.

## Data Availability

The datasets generated/analyzed in preparing the current study are available.

## Supplementary Materials

Supplementary Materials Figure S1: the transfection efficiency of miR-155-5p in neurons was detected by RT-qPCR.
