# Supplementary material for: miR-155-5p in Extracellular Vesicles Derived from Choroid Plexus Epithelial Cells Promotes Autophagy and Inflammation to Aggravate Ischemic Brain Injury in Mice
Source: Oxid Med Cell Longev. 2022 Feb 16;2022:8603427. doi: 10.1155/2022/8603427 (PMC8865969; doi:10.1155/2022/8603427)
Supplement: Supplementary Materials — Figure S1: the transfection efficiency of miR-155-5p in neurons was detected by RT-qPCR. [file 8603427.f1.pdf]

Neurons were transfected with miR-155-5p mimic/inhibitor. We found that in miR-155-5p mimic transfected neurons, miR-155-5p expression was elevated, and in miR-155-5p inhibitor transfected neurons, miR-155-5p expression was decreased.


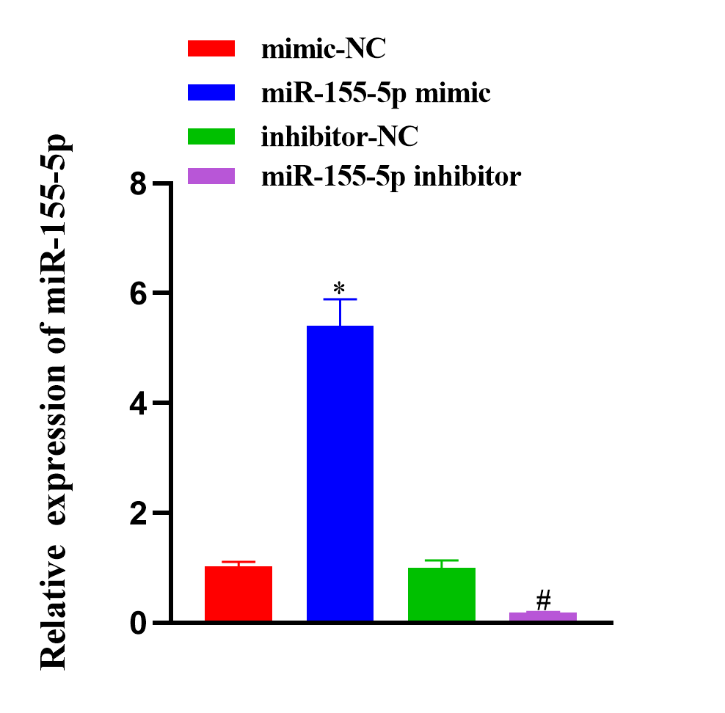


The transfection efficiency of miR-155-5p in neurons was detected by RT-qPCR.
